# Evaluation of the performance of a high-resolution time-of-flight PET system dedicated to the head and breast according to NEMA NU 2-2012 standard

**DOI:** 10.1186/s40658-022-00518-3

**Published:** 2022-12-16

**Authors:** Daisuke Morimoto-Ishikawa, Kohei Hanaoka, Shota Watanabe, Takahiro Yamada, Yoshiyuki Yamakawa, Suzuka Minagawa, Shiho Takenouchi, Atsushi Ohtani, Tetsuro Mizuta, Hayato Kaida, Kazunari Ishii

**Affiliations:** 1 Division of Positron Emission Tomography, Institute of Advanced Clinical Medicine, Kindai University Hospital, 377-2 Ohno-Higashi, Osakasayama, Osaka 589-8511 Japan; 2 Medical Systems Division, Shimadzu Corporation, Kyoto, Japan; 3 Department of Radiology, Kindai University Faculty of Medicine, Osakasayama, Japan

**Keywords:** High resolution, Dedicated head PET, Dedicated breast PET, NEMA, Performance evaluation

## Abstract

**Background:**

This study evaluated the physical performance of a positron emission tomography (PET) system dedicated to the head and breast according to the National Electrical Manufacturers Association (NEMA) NU2-2012 standard.

**Methods:**

The spatial resolution, sensitivity, scatter fraction, count rate characteristics, corrections for count losses and randoms, and image quality of the system were determined. All measurements were performed according to the NEMA NU2-2012 acquisition protocols, but image quality was assessed using a brain-sized phantom. Furthermore, scans of the three-dimensional (3D) Hoffmann brain phantom and mini-Derenzo phantom were acquired to allow visual evaluation of the imaging performance for small structures.

**Results:**

The tangential, radial, and axial full width at half maximum (FWHM) at a 10-mm offset in half the axial field of view were measured as 2.3, 2.5, and 2.9 mm, respectively. The average system sensitivity at the center of the field of view and at a 10-cm radial offset was 7.18 and 8.65 cps/kBq, respectively. The peak noise-equivalent counting rate was 35.2 kcps at 4.8 kBq/ml. The corresponding scatter fraction at the peak noise-equivalent counting rate was 46.8%. The peak true rate and scatter fraction at 8.6 kBq/ml were 127.8 kcps and 54.3%, respectively. The percent contrast value for a 10-mm sphere was approximately 50%. On the 3D Hoffman brain phantom image, the structures of the thin layers composing the phantom were visualized on the sagittal and coronal images. On the mini-Derenzo phantom, each of the 1.6-mm rods was clearly visualized.

**Conclusion:**

Taken together, these results indicate that the head- and breast-dedicated PET system has high resolution and is well suited for clinical PET imaging.

## Background

Positron emission tomography (PET) is a noninvasive imaging method used to visualize and quantify functional biologic processes in living organisms. Through combination with computed tomography (CT), PET imaging has become a routine clinical diagnostic tool in oncology, neurology, and cardiology [1]. With the development of PET detector technology, the traditional photomultiplier tubes have been replaced by avalanche photodiodes or silicon photomultipliers (SiPMs), which can provide timing resolutions below 400 ps [2], and have dramatically improved the image quality of whole-body PET/CT systems [3–5].

While most PET systems are designed to allow screening of the whole body, both brain and breast regional PET imaging plays important roles in many fields, including in clinical use and molecular imaging research [6–8], and there is a high demand for PET systems with high-resolution and high-sensitivity for imaging these sites [9, 10]. However, the spatial resolution of whole-body PET/CT systems is limited by the large gantry aperture that is necessary for a whole-body examination. The limitations imposed by this factor could be reduced in a dedicated scanner for just these parts of the body. The scintillation pixel size is much more relevant for the limitation of the spatial resolution. Gantry size only starts to play a role, when the pixel size is smaller than 2 mm due to residual non-collinearity of the gamma photons. The dedicated brain and breast PET system developed by Shimadzu Co. is a fully tomographic system designed to provide high-resolution and high-sensitivity images through the use of small crystals and a small aperture diameter to enable proximity imaging. In this work, we evaluated the performance of this dedicated PET system according to the National Electrical Manufacturers Association (NEMA) NU2-2012 standards [11], although we partially modified the measurement methods by introducing a brain-sized cylindrical phantom [12]. We discuss the features of the scanner in comparison with conventional whole-body PET/CT systems and assess the imaging performance for detailed structure using imaging of a three-dimensional (3D) Hoffman brain phantom and mini-Derenzo phantom.

## Materials and methods

### System description

**Fig. 1:**
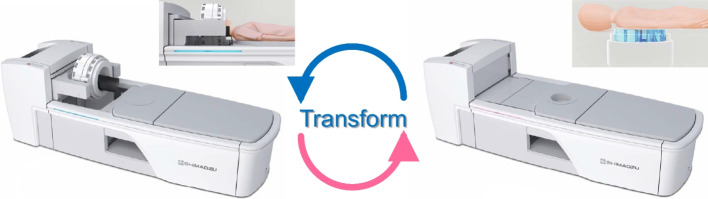
Dedicated brain and breast PET system designed to switch between head scan and breast mode positions

The time-of-flight (TOF) PET system used (SET-5002, a prototype of BresTome™, Shimadzu Corporation, Kyoto, Japan) had an axial field of view (AFOV) of 162 mm that was sufficient to allow whole brain and breast scanning (Fig. 1; URL, https://www.shimadzu.com/med/products/pet/brestome.html). The scanner consists of three detector rings of 300 mm diameter, with each ring consisting of 16 detector modules. Each detector module is in coincidence with the opposing fifteen modules to provide an effective transaxial FOV of more than 268 mm. Each detector module consists of a single layer of LGSO (Lu1.8Gd0.2SiO5:Ce) crystals optically coupled to a SiPM (Multi-Pixel Photon Counter, S14161-3050HS-04, Hamamatsu Photonics KK, Hamamatsu, Japan). The small crystal elements with a size of 2.1 (X) × 2.1 (Y) × 15 mm (depth) are arranged in a 24 (X) × 24 (Y) array. During data acquisition, both prompt and delayed events are saved in list-mode format, and a 3D image is reconstructed at an isotropic voxel size of 1.1 mm with a matrix size of 240 (X) × 240 (Y) × 148 (Z). Attenuation and scatter correction are performed with a modified maximum-likelihood attenuation correction factor and single scatter simulation without external radiation exposure [13].

### Spatial resolution

To evaluate spatial resolution, imaging of a point source inside a capillary tube with an axial extent of less than 1 mm was acquired. The total activity was 1.0 MBq of ^18^F-FDG at the start of the first acquisition. Four points were measured in total, with the source positioned with 1 cm and 10 cm transverse offset, at the center, and at the 1/8 position in the axial direction. Scan durations were determined so that they met the NEMA standards. The acquired data were reconstructed using filtered back-projection after single-slice rebinning with random correction, without corrections for count losses and decay. For each reconstructed images, we obtained full width at half maximum (FWHM) of the transaxial profiles using the maximum intensities of the transaxial image pixels in the radial and tangential directions.

### Sensitivity

Volume sensitivity was measured using five concentric aluminum sleeves of 70 cm in length. ^18^F solution with an activity of 4.70 MBq at the start of scanning was placed inside the aluminum sleeves. The source was placed parallel to the axis of the cylinder of the detector ring, at the center of the transaxial FOV, and at a 10-cm radial distance. Five measurements were performed at each of the radial positions while reducing the number of aluminum sleeves. System sensitivity was calculated as the rate in counts per second that true coincidence events were detected for a given source strength, according to NEMA NU2-2012, section 5.

### Scatter fraction and count rate characteristics

A solid cylindrical phantom composed of polyethylene with a specific gravity of 0.96 with a diameter of 200 mm and length of 700 mm was used to measure scatter fraction and count rate characteristics. An offset hole parallel to the axis was drilled 45 mm away from the axis. A 750-mm-long plastic tube filled with 194.7 MBq of ^18^F was inserted into holes in the phantom that were centered in the transverse and axial directions. Imaging data were acquired for 13.25 h and converted to 117 frames of dynamic 2D sinograms (72 frames of 600 s in addition to 45 frames of 100 s), and the true, scatter, and random count rates were calculated for each frame.

The true, scatter, random, and noise-equivalent count rates (NECRs) were plotted as a function of the effective activity concentration. The NECR was calculated using the following equation:

$$NECR = \frac{{T^{2} }}{T + S + 2R}$$

where T, S, and R are the true, scatter, and random coincidence count rates, respectively.

### Corrections for count losses and randoms

The scatter fraction and count rate characteristics data acquired in the previous section were used to evaluate the accuracy of the corrections for count losses and randoms. The acquired data were reconstructed with corrections for count losses and randoms, within the activity concentration range under the peak NECR. A circular region of interest (ROI) of 180 mm in diameter was placed in each slice at the center of the field of view, and relative mean (averaged value for slices), highest (largest value), and lowest (smallest value) were plotted against the activity concentration.

### Image quality

Because the NEMA IEC Body Phantom does not fit into the small aperture of the system, image quality was evaluated using a brain-sized cylindrical phantom containing the same six spheres (10, 13, 17, 22, 28, and 37 mm) as the NEMA IEC Body Phantom [12]. The activity concentrations were 4.11 kBq/ml for the background and 16.6 kBq/ml for the hot spheres (10, 13, 17, and 22 mm) of ^18^F at scan start. The other two spheres (28 and 37 mm) were filled with cold water. Data were acquired for 30 min and were reconstructed with list-mode dynamic row-action maximum-likelihood algorithm [14] (subsets: 200, relaxation parameter β: 200, iterations: 1), and 3 × 3 × 3 of median filter was applied as a post-filter. Attenuation and scatter were corrected with uniform attenuation coefficient image based on phantom geometry.

For the image analysis, a circular region of interest (ROI) was placed on each sphere on the central slice. The circular ROI size was equal to the inner diameter of each sphere. In addition, twelve 10-mm-diameter circular ROIs were placed in the background at a distance of 10 mm from the edge of the phantom but no closer than 10 mm from any of the spheres. Twelve background ROIs were also placed on slices ± 1 cm and ± 2 cm on either side of the central slice, giving a total of 60 background ROIs. The percent contrast for each sphere and the percent background variability were measured as follows:

$$\begin{aligned} & {\text{Percent contrast for hot spheres}} \;\left( {Q_{H, j} } \right) = \frac{{(C_{H, j} /C_{{B, 10 \;{\text{mm}}}} ) - 1}}{{(a_{H} /a_{B} ) - 1}} \times 100 \left( \% \right) \\ & {\text{Percent contrast for cold spheres}}\; \left( {Q_{C, j} } \right) = \left( {1 - \frac{{C_{C, j} }}{{C_{{B, 10\;{\text{mm}}}} }}} \right) \times 100 \left( \% \right) \\ & {\text{Percent background variability}}\; \left( {N_{{10 \;{\text{mm}}}} } \right) = \frac{{SD_{{10\;{\text{mm}}}} }}{{C_{{B, 10\;{\text{mm}}}} }} \times 100 \left( \% \right) \\ \end{aligned}$$

where *C*_*H,j*_ is the average value in the ROI for each *j* (mm) diameter hot sphere, C_*B,*10 mm_ is the mean of the average value in the 60 background ROIs, *C*_*C,j*_ is the average value in the ROI for each cold sphere, $a_{H}$ is the activity concentration in the hot spheres, and $a_{B}$ is the activity concentration in the background. SD_10 mm_ was calculated as follows:

$${\text{SD}}_{{10\;{\text{mm}}}} = \sqrt {\mathop \sum \limits_{K}^{k = 1} \left( {C_{{B, 10\;{\text{mm}}, k}} - C_{{B, 10\;{\text{mm}}}} } \right)^{2} /\left( {K - 1} \right)} , \quad \left( {K = 60} \right)$$

### Phantom imaging

To evaluate the imaging performance for small structures, images of the 3D Hoffman brain phantom and mini-Derenzo phantom (with hot rod parts of 1.2, 1.6, 2.4, 3.2, 4.0, and 4.8 mm) were acquired and evaluated visually. The 3D Hoffman brain phantom data were acquired for 30 min with ^18^F-FDG containing 19.9 MBq at the scan start and were reconstructed using the conditions of 200 subsets, β = 200, and 1 iteration without post-filter. Attenuation and scatter were corrected with uniform attenuation coefficient image based on phantom geometry. The mini-Derenzo phantom data were acquired for 60 min with ^18^F-FDG at 7.6 MBq at the scan start time and were reconstructed using the conditions of 2048 subsets, β = 1000, and 1 iteration without post-filter so as to obtain high-resolution performance. Attenuation and scatter were not corrected.

## Results

### Spatial resolution

The tangential, radial, and axial FWHM at the 1-mm offset in the 1/2 AFOV were measured as 2.3, 2.5, and 2.9 mm, respectively. All values are summarized in Table 1.

**Table 1:** Spatial resolutions at 1/2 and 1/8 axial offset from the center of the AFOV

Axial position	Offset from the center (mm)	FWHM (mm)			FWTM (mm)		
		Tangential	Radial	Axial	Tangential	Radial	Axial
1/2 AFOV	10	2.3	2.5	2.9	4.5	5.3	5.9
	100	2.8	5.9	2.8	5.3	10.8	5.4
1/8 AFOV	10	2.3	2.2	2.4	4.7	5.1	4.9
	100	2.8	5.6	2.7	6.3	8.9	5.4

*AFOV* axial field of view, *FWHM* full width at half maximum, *FWTH* full width at one-tenth of the maximum

### Sensitivity

**Fig. 2:**
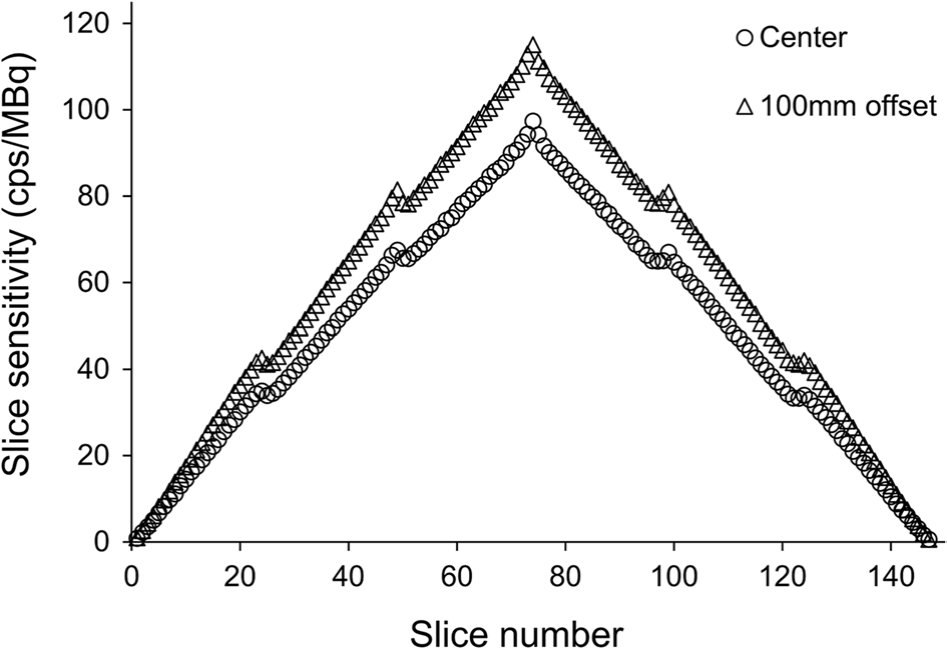
Axial sensitivity profile at the center of the FOV and at 100 mm off-center

The average system sensitivities at the center of the FOV and at a 10-cm radial offset were 7.18 and 8.65 cps/kBq, respectively. The axial sensitivity profiles with the line source placements are shown in Fig. 2, which depicts the sensitivity change along the long axis of the PET system.

### Scatter fraction and count rate characteristics

**Fig. 3:**
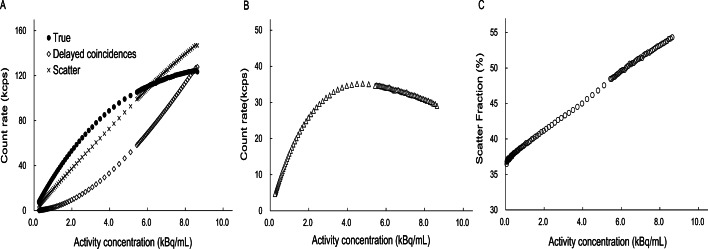
Count rate performance plots. **a** True, delayed coincidences, and scatter count rates. **b** Noise-equivalent count rate as a function of activity concentration. **c** Scatter fraction as a function of activity concentration

Figure 3 shows the count rate performance. The peak noise-equivalent counting rate was 35.2 kcps at 4.8 kBq/ml. The corresponding scatter fraction at the peak noise-equivalent counting rate was 46.8%. The peak true rate and scatter fraction at 8.6 kBq/ml were 127.8 kcps and 54.3%, respectively.

### Corrections for count losses and randoms

**Fig. 4:**
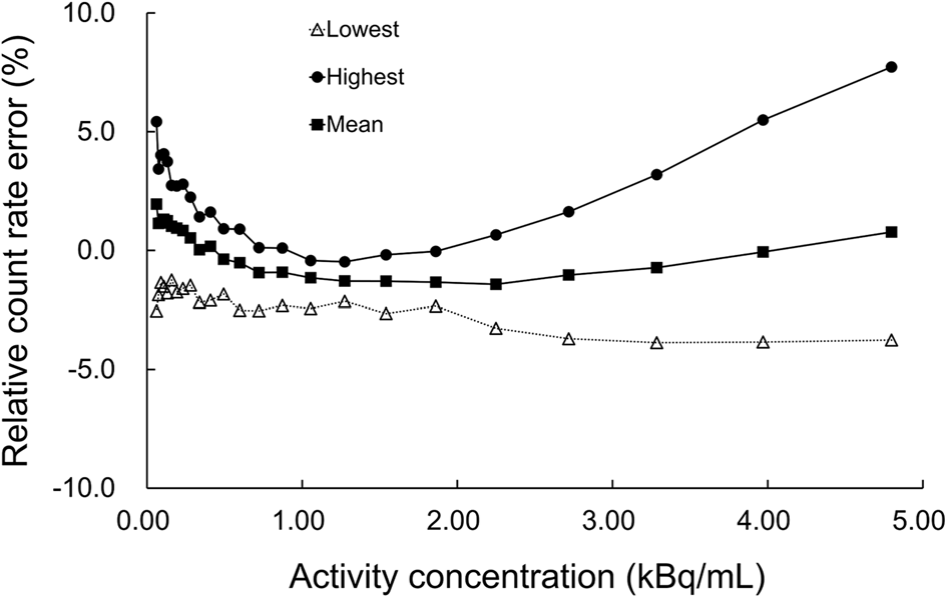
The highest, lowest, and mean values of the bias in relation to activity concentration

Figure 4 shows the highest, lowest, and mean biases as a function of activity concentrations. The maximum absolute bias was 11.5%.

### Image quality

**Fig. 5:**
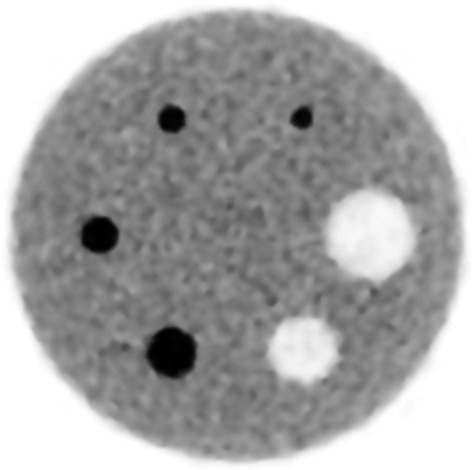
PET image of the brain-size image-quality phantom used to measure percent contrast and percent background variability

The percent contrast and percent background variability are shown in Table 2. Figure 5 shows PET images obtained on the dedicated brain and breast PET system. The hot and cold spheres are clearly visualized.

**Table 2:** Percent contrast and percent background variability evaluated with the brain-size image-quality phantom

Parameter	Sphere diameter (mm)					
	10	13	17	22	28	37
Percent contrast for hot spheres (%)	49.9 ± 23.3	63.2 ± 22.6	72.4 ± 22.0	79.5 ± 21.5	–	–
Percent contrast for cold spheres (%)	–	–	–	–	81.8 ± 6.7	84.8 ± 7.1
Percent background variability (%)	4.2	–	–	–	–	–

Data are presented as mean ± standard deviation

### Phantom imaging

**Fig. 6:**
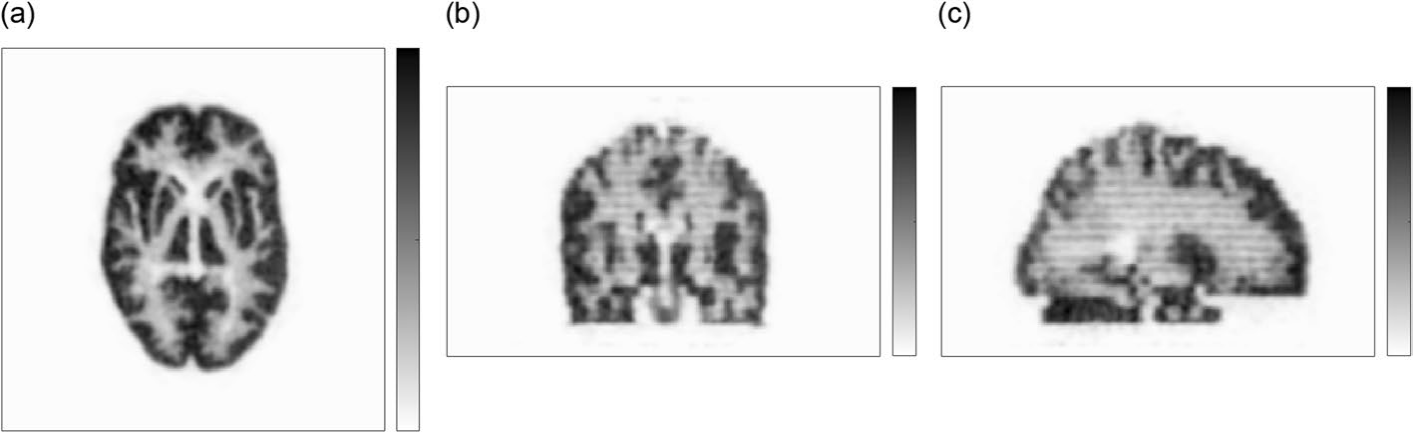
Representative slices of the 3D Hoffman brain phantom image: **a** transaxial, **b** coronal, **c** sagittal. The thin activity distribution in gaps was visible in both coronal and sagittal views

Figure 6 shows an image of the 3D Hoffman brain phantom. The transverse image clearly shows detailed structure of the gray and white matter of the phantom. Furthermore, the structure of the thin gaps that compose the phantom is visible in the sagittal and coronal images.

**Fig. 7:**
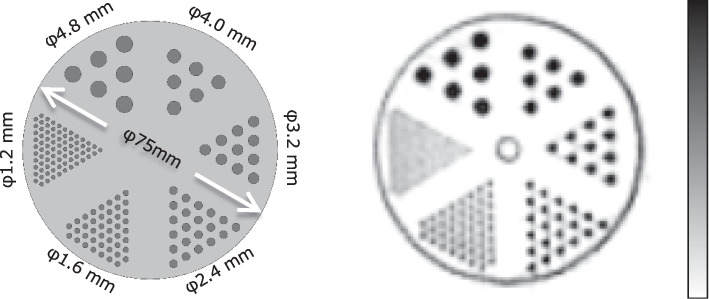
Image of the mini-Derenzo phantom. The original distribution (left) and the obtained image (right). Rods of 1.6 mm and higher are visually separated

Figure 7 shows a mini-Derenzo phantom image averaged 28 slices in axial direction. Each of the 1.6-mm rods is clearly visualized.

## Discussion

We evaluated the physical performance of the dedicated brain and breast PET system according to spatial resolution, sensitivity, count rate, accuracy of count loss, and image quality, in accord with the NEMA NU2-2012 standard. Our results provide a reference on the imaging system characteristics and allow comparisons with other commercial PET systems.

### Spatial resolution

Although the development of head-specific PET systems has been active in recent years, the only internationally standardized guideline for human PET performance evaluation is NEMA NU 2, and because there are evaluation items that cannot be performed due to limitations in scanner geometric conditions, many dedicated head PET systems are evaluated using NU 4 or other proprietary methods. Therefore, the results of the performance evaluation of the dedicated head PET cannot be compared uniformly. However, the spatial resolution obtained from the FBP reconstructed image is considered comparable as a reference value. The FWHM of other dedicated head PET systems [15–18] generally achieves a FWHM in the latter half of 1 mm to 3 mm, and this system is comparable to those systems. However, we believe that the spatial resolution in clinical use should be evaluated based on the difference in the ability to render structures in phantoms with complex distribution shapes.

The dedicated PET system can achieve better spatial resolution than whole-body PET/CT systems [3–5]. The improvement can be explained by the smaller crystal size and the suppression of non-collinearity with its smaller diameter.

From axial offset of 10 to 100 mm, the spatial resolution of the PET system was degraded by approximately 2.4 times, which is numerically higher than that obtained with other systems [3–5]. The incident angle of the radial direction in the 100-mm offset position in the dedicated system, which has a small detector ring diameter, is larger than that of whole-body PET systems, resulting in an increase in parallax error. This is reasonable considering that filtered back-projection reconstruction was performed. At 100-mm offset, the position of the source in this system was outside of 70% of the effective FOV, which means that most of the imaging target would be imaged inside of this. However, iterative reconstruction algorithms are commonly used in clinical practice, and taking into account these factors, no adverse effects are expected. Although the depth of interaction measurement is theoretically effective for suppressing parallax errors in small aperture systems, this system consists of both SiPMs and small crystals to improve the crystal discrimination and obtain effective TOF gain for image reconstruction. Because of the advantage of the detector module, in standard clinical reconstruction settings it should be feasible for this system to achieve spatial resolution equivalent to that of conventional dedicated breast PET systems with depth of interaction [19]. Although the advantage of configuration of SiPMs and small crystals was not reflected in the results of the NEMA performance evaluations, we believe that these effects are represented in the mini-Derenzo phantom images, as described below.

### Sensitivity

The four discontinuities in the axial profile of sensitivity are due to the effect of the zero count of the gap between detector rings. The detector modules of this system were arranged in the axis direction with 3 modules separated by a 1-crystal gap (virtual detector). Since no events were detected in the virtual detector, the counts of the line of responses paired with the virtual detector were zero, and single-slice rebinning resulted in a step-like profile. The sensitivity of this system at the 100-mm radial offset is approximately 1.2 times higher than at the center, although the sensitivity at the center of the transaxial FOV and at a 100-mm radial distance provided similar values to many whole-body PET/CT system performance evaluations [3–5]. Line of responses passing away from the center of the FOV are farther away from some detectors but closer to many other detectors, while line of responses passing through the center of the FOV are geometrically the farthest from all detectors. Hence, the measurement with offset position resulted in higher sensitivity, with the effect generally increasing with decreasing detector ring diameter.

### Scatter fraction and count rate characteristics

Both the NECR peak and its activity concentration are lower with the dedicated PET system than with any other PET/CT system, with peak NECR occurring at activity concentrations of approximately 20–30 kBq/mL (124.4 kcps at 18.85 kBq/mL for the uMI550, 305kcps at 32.6 kBq/mL for the Vision, and 153.4 kcps at 54.9 kBq/mL for the Veros) [3–5]. In radiation measurement, the single count rate is inversely proportional to the square of the distance between the radiation source and the detector, so that for the same source distribution, the single count rate is higher for a PET system with a smaller aperture, and the dead time and random coincidence count rates increase remarkably. In most clinical examinations, activity concentrations of approximately 2–4 kBq/mL are present, levels that are consistent with our results on sensitivity performance. We expect that image quality relative to the distribution of radioactivity in the body can be obtained with less dependence on NECR. About scatter fraction, the smaller the detector diameter, the closer the line of response of the scattered coincidence is to the true distribution. Therefore, higher scatter fraction was considered reasonable because the scattering component included in the range of ± 12 cm increased in the NEMA analysis.

### Corrections for count losses and randoms

Although a slight bias was observed in the low-activity region as a Lu background, the relative counting rate errors were shown to be sufficiently small for clinically relevant activity levels, as described in the previous section. The radiation activity in the subject matter is quite different between brain PET and breast PET, because brain PET scans are performed independently using a variety of tracers, whereas dedicated breast PET scans are usually performed after a whole-body PET/CT scan. In the expected radiation activity range in clinical situations, this system showed a proper correction of dead time losses and random events.

### Image quality

Since the IEC Body Phantom could not be placed in the field of view because it was larger than the aperture of this device, we used a phantom with a smaller outer diameter than the IEC Body Phantom but the same internal sphere size as the IEC Body Phantom and no lung inserts [12]. The phantom we used tends to reduce attenuation and scattering compared to the IEC Body Phantom, resulting in better contrast and noise values. We believe that this was a feasible and appropriate image-quality evaluation method for this system, as it reflected image quality under conditions close to those of the head and breast, which were the imaging targets of this system. The percent contrast value of approximately 50% for the 10-mm spheres did not provide an outstanding advantage over other PET/CT systems [3–5]. However, given the hot contrast results for 10-, 13-, 17-, and 22-mm spheres, which increased as the diameter increased, this reasonable property of partial volume effect may increase the visibility of fine structures, contribute to the evaluation of intratumoral morphologic and metabolic characteristics, and improve the quantification of ^18^F-FDG uptake.

### Phantom imaging

Since the performance of this PET system as a PET scanner does not change when the imaging mode is changed, the results of both the Hoffman Phantom and Mini-Derenzo Phantom show the imaging performance provided in both modes.

The 3D Hoffman brain phantom has gaps thinner than the common spatial resolution of PET/CT systems, so as to provide an activity ratio of 1:4 for white matter to gray matter. These bars showed that the radioactivity distributed in the thin gaps, which were not visualized by the whole-body PET/CT systems, appeared as true distribution. Using the mini-Derenzo phantom, rods of 1.6 mm diameter can just be distinguished by the eye, indicating that high-resolution performance equivalent to that of small animal PET systems can be obtained [20]. This result indicates that the spatial resolution was higher than that required by the NEMA NU2-2012 procedures for spatial resolution.

### Limitations

The PET system performance should be evaluated to ensure it conforms with the NEMA standard. However, we partially changed the NEMA NU 2–2012 standards to make them applicable to the performance evaluation of this dedicated PET system. The NEMA IEC Body Phantom is designed for use with whole-body cylindrical PET/CT systems and was too big to be inserted into the space available in the dedicated PET system. We evaluated image quality by changing only the outer cylindrical container without modifying the six spheres. This change led to scatterers of different sizes, which did not allow for an accurate comparison with whole-body PET/CT systems. However, we believe that our system with TOF is sufficient to examine the trend of image quality because it can reduce the effect of the difference in scatterer size.

## Conclusions

According to the present assessment based on the NEMA NU 2–2012 standards, the PET detector dedicated head and breast has excellent high spatial resolution. The phantom scans demonstrate that the high spatial resolution of this PET detector has the potential to improve the imaging of small and/or low contrast lesions.

## Data Availability

The datasets used and/or analyzed during the current study are available from the corresponding author on reasonable request.

## Abbreviations

PET: Positron emission tomography
CT: Computed tomography
SiPM: Silicon photomultiplier
NEMA: National Electrical Manufacturers Association
3D: Three-dimensional
TOF: Time-of-flight
FOV: Field of view
FWHM: Full width at half maximum
NECR: Noise-equivalent count rate
ROI: Region of interest
